# Comparing two different modes of mechanical ventilation by the least square fitting method: nava versus PSV

**DOI:** 10.1186/2197-425X-3-S1-A319

**Published:** 2015-10-01

**Authors:** S Spadaro, S Grasso, V Cricca, F Dalla Corte, R Di Mussi, G Biondi, G Valpiani, S Zardi, A Romanello, E Marangoni, CA Volta

**Affiliations:** University of Ferrara / Intensive Care Unit, Morphology Surgery and Experimental Medicine, Ferrara, Italy; University of Foggia, Medicina Sperimentale e Rigenerativa, Foggia, Italy; Dipartimento di Emergenza e Trapianti d'Organo, University of Bari, Bari, Italy

## Introduction

The Least Square Fitting (LSF) method is a statistical approach used for evaluating respiratory mechanics [1]. It allows measurement of respiratory mechanics continuously at the bedside, even in presence of flow limitation [23], without the need for constant inspiratory flow rate, end-inspiratory hold and end-expiratory occlusion. These features allow the application of the LSF method to assisted ventilation modes, such as pressure support ventilation (PSV) [3] and neurally-adjusted ventilatory assist (NAVA).

## Objectives

We compared the LSF performance during PSV and NAVA. Our hypothesis was that the LSF works better during NAVA than during PSV, since NAVA algorithm allows a more accurate neuro-ventilatory coupling.

## Methods

15 patients undergoing mechanical ventilation for acute respiratory failure were ventilated using randomly either PSV or NAVA. Data of resistance (Rrs), elastance (Ers) and total positive end expiratory pressure (PEEPtot) were obtained by fitting the equation Paw = Rrs x V´ + V_T_/Crs + PEEPtot during inspiration. The coefficient of determination (CD) of the applied equation was used to compare data obtained during NAVA and PSV, the higher being the CD, the better the quality of the data. These data were obtained at the beginning of mechanical ventilation (T0), and after 12 (T12), 24 (T24), 36 (T36), 48 (T48), 60 (T60) and 72 (T72) hours of mechanical ventilation.

## Results

Data obtained with LSF were statistically more reliable during NAVA than during PSV (Chi-squared test: p < 0.001). The CD level showed a higher value during NAVA (T0 median 0.9855), that was maintained constantly higher in time, than during PSV (T0 median 0.9288), in which the value of the CD progressively worsened by the hours of mechanical ventilation.

## Conclusions

The LSF method of the LSF performs better during NAVA then during PSV. By the hours of mechanical ventilation the performance of the LSF method further worsens during PSV while remains constant during NAVA. Our data indirectly confirm more physiological patient-ventilation interactions during NAVA than during PSV.

**Figure 1:**
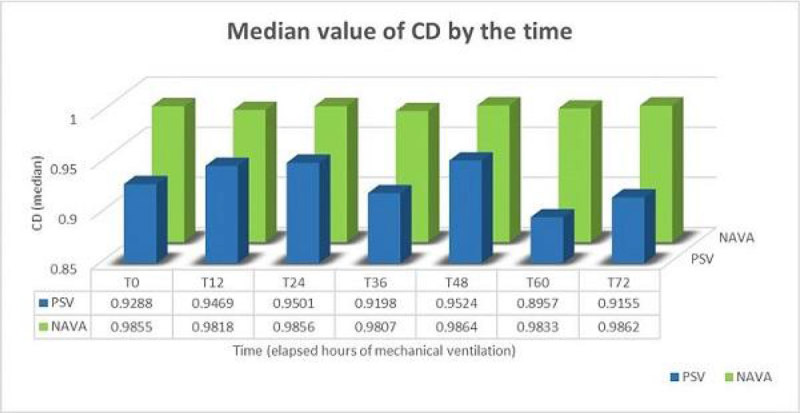
Figure 1

**Figure 2:**
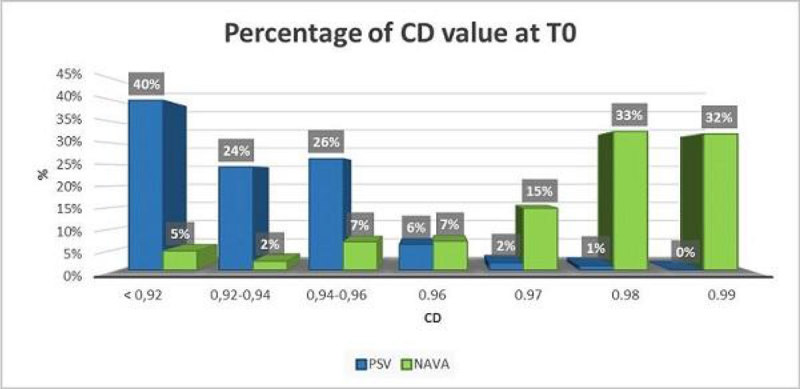
Figure 2

**Table 1:** Table 1

Pt	Sex (M:F)	Age (years)	Height (cm)	Weight (kg)	Pathology	MV mode
1	F	74	154	90	Septic shock	PSV
2	F	54	160	77	Postoperative respiratory failure	PSV
3	M	81	172	70	Heart failure	PSV
4	M	66	175	75	Hemorrhagic shock	PSV
5	M	74	178	120	Heart failure	PSV
6	M	73	175	85	Septic shock	PSV
7	M	38	180	80	Thoracic and abdominal trauma	PSV
8	M	78	174	70	Septic shock	PSV
Mean	6:2	67 ± 15	171 ± 9	83 ± 16		

**Table 2:** Table 2

Pt	Sex (M:F)	Age (years)	Height (cm)	Weight (kg)	Pathology	MV mode
9	F	58	160	56	Septic shock	NAVA
10	F	80	158	70	Heart failure	NAVA
11	F	81	170	76	Acute exacerbation of COPD	NAVA
12	F	63	170	85	ARDS	NAVA
13	M	64	165	75	Acute hypertensive pulmonary edema	NAVA
14	F	78	160	65	Septic shock	NAVA
15	F	68	168	72	ARDS	NAVA
Mean	1:6	70 ± 9	164 ± 5	71 ± 9		
